# Proteomic analysis of Cucumis sativus cotyledons after glucohexaose treatment as a part of ROS accumulation related resistance mechanism

**DOI:** 10.1186/1477-5956-12-34

**Published:** 2014-06-17

**Authors:** Yuhan Hao, Chunmei Lin, Haiyan Fan, Yang Yu, Ning Li, Shaoli Chen

**Affiliations:** 1 College of Bioscience and Biotechnology, Shenyang Agricultural University, Shenyang 110866, PR China; 2 Key Laboratory of Protected Horticulture of Ministry of Education, Shenyang Agricultural University, Shenyang 110866, PR China; 3 State Key Laboratory of Genetic Engineering and Institute of Plant Biology, School of Life Sciences, Fudan University, Shanghai 200433, PR China; 4 Fruit and Silkworm Administrative Station of Liaoning Province, Shenyang 110866, PR China

**Keywords:** *Cucumis sativus*, Glucohexaose, Reactive oxygen species, Proteome

## Abstract

**Background:**

Glucohexaose is a safe farm chemical used for pathogen prevention, which can induce systemic acquired resistance in cucumber.

**Results:**

We found that glucohexaose treatment of cucumber plant induced an accumulation of the reactive oxidative species (ROS). Histochemistry showed sharp increases in O^2-^ and H_2_O_2_ 5 h after glucohexaose treatment. After 5 h, the O^2-^ content decreased to a normal level, but the H_2_O_2_ content remained at a high level 10 h after glucohexaose treatment. And antioxidant enzymes were also changed after glucohexaose treatment. We also investigated the relationship between ROS accumulation and glucohexaose-induced proteome alteration using 2D electrophoresis coupled with MS/MS. 54 protein spots, which enhanced expression under glucohexaose treatment but suppressed the expression by application of DPI and DMTU, have been identified.

**Conclusion:**

Our study showed the accumulation of ROS is a part of mechanism of glucohexaose induced resistance in cucumber cotyledons. The up-regulated proteins identified by MS such as PP2C and antioxidation proteins are important in ROS signaling. It will be interesting to find out the regulatory mechanism underlying the induction of these proteins via ROS, and provide some clues to the mechanism of glucohexaose-induced resistance.

## Introduction

During vegetable production, chemical pesticides are still the main method of disease prevention. As consumers’ concerns about food quality increase, how to prevent disease without pesticide residues has attracted more attention. Induced resistance by biotic and abiotic elicitors is a new method for disease resistance. Glucohexaose, synthesized by the Research Center for Eco-Environmental Science, Chinese Academy of Science, is a safe, synthetic oligosaccharide elicitor that is naturally degraded in the environment. After glucohexaose incubation, plant resistance systems were activated and plants acquired stronger resistance to many pathogens, such as *Pseudoperonospora cubensis*[1]. Using glucohexaose in agricultural production would be a safer and more acceptable alternative to chemical pesticides. However, the mechanism of the induced resistance remains unclear. A previous proteomic study in our laboratory using cucumber leaves after glucohexaose treatment identified certain ROS accumulation related proteins [2], which indicated that the ROS accumulation might be one of the mechanisms of glucohexaose-induced resistance.

ROS, including superoxide (O^2-^), hydrogen peroxide (H_2_O_2_), hydroxyl radical (OH·) and singlet oxygen (^1^O_2_), play an important role in resistance to pathogens as signal molecules in plant cells [3]. The oxidative burst (OXB) was first reported by Doke in 1983 in a study of the interaction between potato tuber tissues and *Phytophthora infestan*[4], which involves the rapid release of ROS in the early stage of pathogen infection. Later studies indicated that in many stress conditions, such as bacterial, viral and fungal infection, the induction of elicitors and the composition of the cell wall, and mechanical stress could lead to the rapid release of ROS [5,6]. The main ROS in plant cells is H_2_O_2_, which can be transported across cell membranes to act as a signal molecule and is an essential signaling mediator of plant stress resistance [7-9]. There are different ROS generating mechanisms in different plants, such as germin-like oxalate oxidase, polyamine oxidase, peroxidase, thioredoxins and glutaredoxins [10-16]. However, in most plants, NADPH oxidase is the principal source of ROS induced by pathogens or elicitors [17,18]. NADPH oxidase, located in the cell membrane, is a redox enzyme containing a heme moiety and six transmembrane domains. It transfers an electron from NADPH to O_2_, and generates large amounts of O^2-^ in a short time [18].

Proteomic study is a good tool to investigate the mechanism of ROS related glucohexaose induced resistance. It is used in other stress related ROS pathway studies in different species. Soares, et al. investigated wound related proteome changes in ROS pathway in *Medicago* and found some interesting proteins such as SODs, peroxidases and germin-like proteins [19]. Wang, et al. found AtCIAPIN1 and flg22 are early-responsive redox-sensitive proteins in *Arabidopsis* with proteomic studies [20]. In wheat, Bykova, et al. reported several redox-sensitive proteins functioning in seed dormancy control [21]. Therefore, we sought to use proteomic tool to further investigate the possible link between glucohexaose-induced resistance and ROS accumulation. We report that glucohexaose can induce ROS accumulation in cucumber cotyledons and provide some clues concerning the mechanism of glucohexaose-induced ROS accumulation. These results provide a theoretical basis for developing safe farm chemicals for vegetable production.

## Materials and methods

### Plant materials

Cucumber seeds (Jinyan No. 4) were soaked in water for 24 h and then sterilized with 75% ethanol for 30 s and 2.5% NaClO for 15 min. After washing with sterile water at least three times, sterilized seeds were placed on sterile water soaked gauze. The seeds were allowed to germinate at 25–30°C. When the cotyledons expanded, the seedlings were used for subsequent experiments.

### The detection of variation of H_2_O_2_ and O^2-^ in glucohexaose-treated cotyledons

Whole plants were sprayed with 50 μg/ml glucohexaose and H_2_O_2_ and O^2-^ were detected at 1, 2, 3, 4, 5, 6, 7, 8, 9, 10, 11, 12, 13, 14 and 15 h after glucohexaose treatment. We detected H_2_O_2_ and O^2-^ using DAB and NBT staining methods, according to Zhang et al. [22] and Soares et al. [19] with modifications. Cucumber cotyledons were soaked with 1 mg/ml DAB (Sigma, St. Louis, MO, USA) for 8 h and infiltrated with 0.1% NBT (Ameresco, OH, USA) for 20 min, respectively. The cotyledons were then transferred to 95% ethanol in an 80°C water bath. After the green color of the cotyledons disappeared, the cotyledons were photographed to show the variation of H_2_O_2_ and O^2-^. Cotyledons were preserved in 95% ethanol at 4°C. Three independent replicates preformed for each assay.

To investigate the effect of DPI (an inhibitor of NADPH oxidase) and DMTU (a ROS scavenger) during the oxidative burst, we treated two groups of plants with glucohexaose after incubating them with 100 μM DPI and 5 mM DMTU for 4 h.

### Determination of scavenger enzymes activity

Assay kits (Nanjing Jiancheng Bioengineering Institute, China) were used to measure SOD activity, MDA contents, POD activity, CAT activity, APX activity and GPX activity.

### Protein extraction

Proteins were extracted with a PEG precipitation method according to Xi et al. [23], with modifications. Cucumber cotyledons were collected and pulverized to a fine powder with liquid nitrogen. The finely ground powder was extracted with Mg/NP-40 extraction buffer containing 0.5 M Tris–HCl (pH 8.3), 2% (v/v) NP-40, 20 mM MgCl_2_, 2% (v/v) β-mercaptoethanol, 1 mM PMSF, 1% (w/v) PVP and 1 mM EDTA. After centrifugation at 13000 × g for 15 min, the supernatant was precipitated with 50% PEG stock solution to adjust the final PEG concentration to 24%, which is the appropriate PEG concentration for cucumber Rubisco protein precipitation. After centrifugation at 13 000 × g for 30 min, the pellet was named as fraction F1 and the supernatant was precipitated with 10% (TCA)/acetone solution at -20°C for at least 1 h. The TCA/acetone precipitation fraction was centrifuged at 13000 × g for 30 min and the pellet named as fraction F2. The F1 and F2 pellets were washed with TCA/acetone until they were colorless, and then they were washed three times with 80% acetone containing 0.07% β-mercaptoethanol. Proteins were freeze-dried and stored at -80°C for subsequent tests.

### 2-D electrophoresis

The dried proteins were redissolved in lysis buffer containing 8 M Urea, 2 M Thiourea, 4% CHAPS, 1% DTT, 1% TBP and 2% IPG buffer for 3–4 h at 30°C. The samples were centrifuged at 12000 × g for 10 min at room temperature and the pellet was discarded. The supernatant was tested by the Bradford method to determine the protein content and loaded onto 24 cm pH 4–7 IPG stripes with 1 mg and 450 μl protein solution. The IEF conditions were as follows: 50 V for 15 h, 100 V for 1 h, 250 V for 3 h, 500 V for 3 h, 1000 V for 1 h, 10000 V for 3 h, 10000 V for 160000 Vh and 500 V thereafter (Ettan IPGphorIII, GE Healthcare). After IEF, the focused strips were equilibrated with equilibration solutions twice. 2% DTT and 2.5% iodoacetamide were added to equilibration mother solution, which contained 6 M Urea, 0.05 M pH 8.8 Tris–HCl, 2% SDS and 20% glycerol. The second dimension SDS-PAGE was performed on an Ettan DALT six (GE Healthcare) with an 11% polyacrylamide gel. CBB R350 was used to stain the 2D gels, according to the operation manual (GE Healthcare).

### Image analysis

A UMAX Power Look 2100XL (Maxium Tech., Taipei, China) was used to scan the 2D gels in TIF images. The spots are then analyzed by PDQuest Advanced™ 2-D Analysis software (version 8.0.1, Bio-Rad). Each image was adjusted to be the same size and the Spot Detection Parameter Wizard was used to automatically pair the spots on each image. Landmarks and manual matching helped the accuracy of the pairing. Quantitative analysis was performed by Student’s test only for notable spots in groups of three biological replicated gels. Protein spots selected for future identification showed an increase of at least 2.0-fold in the P group compared with the CK group, and at the same time the DPI and DMTU group were decreased relative to the P group. Here, CK represents the control group and P represents the cucumber cotyledons treated with 50 μg/ml glucohexaose for 5 h. The DPI and DMTU groups represent those treated with DPI and DMTU for 4 h before 50 μg/ml glucohexaose treatment.

### Protein identification by MS

The selected spots were excised from gels using pipette tips, placed in tubes and decolored using 200–400 μl 100 mM NH_4_HCO_3_/30% ACN. After freeze-drying, the protein spots were digested by trypsin (the ratio of trypsin to proteins was 1:20–1:100) for approximately 20 h at 37°C. The hydrolysates were then transferred to new tubes and disrupted by sonication for 15 min in a buffer containing 100 μl 60% ACN/0.1% TFA and desalinated in a Ziptip (Millipore). The in-gel digested proteins were freeze-dried and resolved by 2 μl 20% ACN for each 1 μl of protein. After air-drying, 0.5 μl of over-saturated CHCA solution was added (dissolved in 50% ACN and 0.1% TFA) and the proteins were then air-dried. A 4800 Plus MALDI TOF/TOFTM Analyzer (Applied Biosystems, USA) was used for MS analysis with the Nd:YAG lasing light emitter, 2 kV voltage, positive ion model, 800–4000 Da PMF quality scan range. MS/MS analysis was performed with parent irons with signal to noise ratios of more than 50, and excited 2500 times by the MS/MS laser with a collision energy of 2 kV and CID closure. For database searching, the conditions were set as follows: database: IPI, taxonomy: Viridiplantae (900091), type of search: Peptide Mass Fingerprint (MS/MS Ion Search), enzyme: Trypsin, Fixed modifications: Carbamidomethyl (C), mass values: Monoisotopic, protein mass: Unrestricted, peptide mass tolerance: ±100 ppm, fragment mass tolerance: ±0.8 Da, peptide charge state: 1+ and max missed cleavages: 1.

## Results

### Glucohexaose-induced ROS accumulation

**Figure 1:**
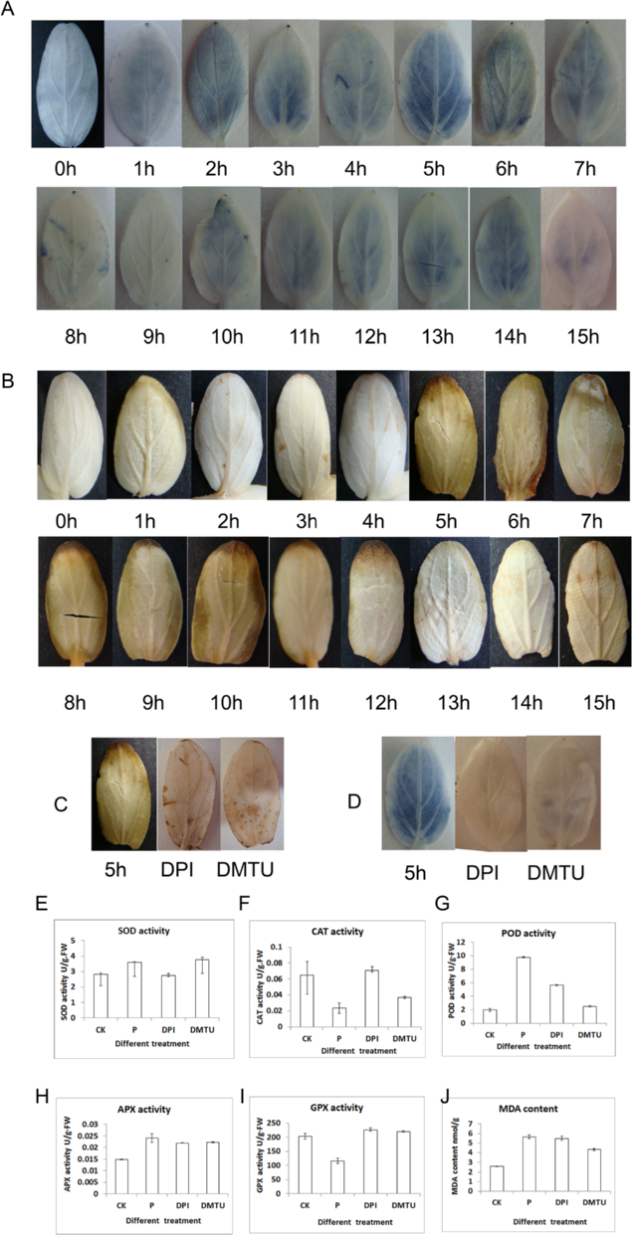
**Glucohexaose can induce ROS accumulation. A-B**. Changes of O^2-^**(A)** and H_2_O_2_**(B)** after treatment of glucohexaose. Cucumber cotyledons are infiltration with 0.1% NBT and 1 mg/mL DAB and decolourisation. One to fifteen hours after glucohexaose treatment indicated the plants’ early respond to glucohexaose. **C-D**. DPI and DMTU incubation can eliminate the ROS accumulation at five hours after glucohexaose treatment. **E-J**. Some important ROS scavenging enzymes activity in cucumber cotyledons with different treatment. CK represent the control group; P represent the cucumber cotyledons treated with 50 μg/mL glucohexaose for five hours; DPI and DMTU represent before treated with 50 μg/mL glucohexaose, DPI and DMTU were incubated for four hours. Error bar represent SD.

**Figure 2:**
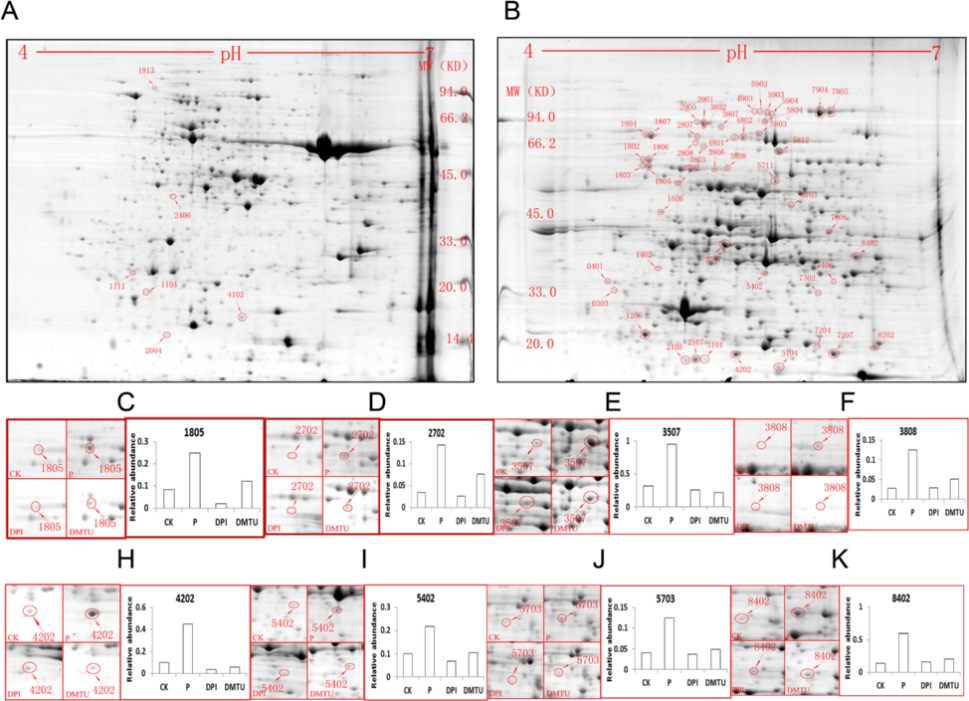
**Proteomic analysis of glucohexaose induced ROS accumulation. A-B** 2D maps of identified differential expression cucumber cotyledon proteins spots. These two maps are from the P group and other groups’ picture offered in supplemental materials. **C-K** Representative differential protein spots and their relative abundance. All identified protein spots’ information is supplied in supplemental materials.

To determine whether glucohexaose can actually induce an ROS accumulation in cucumber cells and when the accumulation occurs, we detected two ROS, H_2_O_2_ and O^2-^, using DAB and NBT staining at 1, 2, 3, 4, 5, 6, 7, 8, 9, 10, 11, 12, 13, 14 and 15 h after glucohexaose treatment. H_2_O_2_ and O^2-^ began to accumulate after glucohexaose treatment and peaked at 5 h after treatment (Figure 1A and B). After incubated with the inhibitor of NADPH oxidase DPI and the ROS scavenger DMTU before glucohexaose treatment, ROS failed to accumulate (Figure 1C and D). Antioxidant enzymes were also changed after glucohexaose treatment. SOD activity indicated O^2-^ level increased after glucohexaose treatment (Figure 1E). As a main signaling messenger, H_2_O_2_ scavengers behaved differently: POD and APX activities increased and CAT and GPX activities decreased after glucohexaose treatment (Figure 2E-J). The different responses of the four H_2_O_2_ scavengers indicated a complicated mechanism of H_2_O_2_ regulation in the plant cells. Moreover, SOD, CAT, POD, APX and GPX activity returned to the control level after DPI and DMTU incubation. These results showed that ROS accumulated at 5 h after glucohexaose treatment. As ROS accumulation is an important part of plant immune action, it is also a part of mechanism of glucohexaose induced cucumber resistance.

### Proteomic analysis of cucumber cotyledons after ROS accumulation induced by glucohexaose

Because H_2_O_2_ and O^2-^ showed sharp increases at 5 h after glucohexaose treatment, however, DMTU or DPI pretreatment significantly abolished the effect of glucohexaose. To find out the regulatory mechanism underlying the induction of proteins via ROS, four samples were applied to 2-DE analysis. At first, sample 1 and 2 were sprayed with deionized water, sample 3 and 4 were pretreated with 5 μM DMTU and 100 μM DPI, respectively. After 4 h of pretreatment, deionized water was used to spray sample 1 (control plants), sample 2, 3, 4 were sprayed with 50 μg/ml glucohexaose. Then, at 5 h after last treatment, the cucumber cotyledons were harvested.

Total proteins extracted from cucumber cotyledons were divided into two fractions, F1 and F2, using the PEG precipitation method. After 2D electrophoresis and image analysis by MS, we identified 55 protein spots that showed significant expression changes. Fifty-four protein spots (Figure 2, Additional file 1: Figure S1 and Additional file 1: Figure S2) corresponding to 37 proteins were identified by MALDI-TOF-TOF MS (Table 1). The protein spots chosen showed increased abundance after glucohexaose treatment and decreased when incubated with DPI or DMTU; therefore, they are possibly related to the glucohexaose induced ROS accumulation. After data analysis, sequence alignment, GO annotation and reference searching, we divided these proteins into eight groups: photosynthesis-related proteins, respiration and metabolism-related proteins, translation-related proteins, proteolytic enzymes, protein phosphatases, antioxidation proteins and unclassified proteins. Each group may play an important role in glucohexaose induced ROS accumulation.

**Table 1:** Glucohxaose and ROS modulators-regulated proteins

**ID**	**gi**	**Protein name**	**PI**	**MW**	**Protein score**	**Protein score C.I. %**	**Pep. count**	**E-value**	**Fold enhancement by P6**	**Percentage of suppression by DPI/DMTU**
**Photosynthesis related proteins**										
1104	gi|12620881	Ribulose-1,5-bisphosphate carboxylase/oxygenase activase	5.54	48186.1	284	100	9		1.68	84.15/61.03
1111	gi|115768	Chlorophyll a-b binding protein of LHCII type I	5.14	27331.7	282	100	7		2.05	69.18/52.48
2004	gi|325515965	Ribulose-1,5-bisphosphate carboxylase/oxygenase large subunit	6.67	23461.8	651	100	12		7.76	50.81/24.37
4102	gi|255567170	Chlorophyll A/B binding protein	6.85	29362	82	99.482	5		1.40	82.39/55.08
0401	gi|62899808	Chromoplast-specific carotenoid-associated protein	5.05	35272.5	581	100	16		11.35	86.13/83.53
3507	gi|125578	Phosphoribulokinase	6.03	44485.6	552	100	12		2.97	73.35/77.00
3806	gi|12585325	Phosphoglucomutase	5.56	68625.8	322	100	8		2.36	48.99/41.42
5104	gi|11134156	Oxygen-evolving enhancer protein 2	8.61	28292.3	786	100	12		2.57	17.35/36.94
**Metabolism-related proteins**										
1206	gi|225451299	Ribose-5-phosphate isomerase	6.66	30340.1	291	100	7		1.58	49.04/61.65
1606	gi|147838694	Chloroplast fructose-1,6-bisphosphatase	5.3	45183.4	898	100	11		3.55	88.59/33.13
2406	gi|3328122	Phosphoglycerate kinase precursor	7.68	50594	449	100	9		1.87	57.87/50.11
2702	gi|118721470	Vacuolar H + -ATPase subunit B	5.18	54450.9	575	100	17		4.21	81.96/47.07
5402	gi|307136265	Fructokinase	5.61	35800.6	403	100	16		2.15	68.33/52.23
7207	gi| 2833386	Ribulose-phosphate 3-epimerase	8.23	30632.2	299	100	5		9.18	79.61/78.13
7904	gi|1351856	Aconitate hydratase	5.74	98569.8	884	100	23		3.60	90.35/74.12
7905	gi|1351856	Aconitate hydratase	5.74	98569.8	1070	100	26		7.19	91.27/76.31
8402	gi|255557204	Fructose-bisphosphate aldolase, putative	7.59	38745.2	121	100	3		4.44	73.13/66.04
**Chaperones and elongation factors**										
1813	gi|225445166	Elongation factor ts	4.78	123315.5	110	100	6		7.76	53.68/63.39
1804	gi|124245039	Chloroplast HSP70	5.18	75464.1	1070	100	28		15.05	99.55/86.18
1807	gi|124245039	Chloroplast HSP70	5.18	75464.1	1050	100	27		12.82	97.76/79.11
2808	gi|6911551	Heat shock protein 70	5.07	71843.3	570	100	25		5.65	95.26/76.54
3101	gi|255550363	Groes chaperonin	8.89	26582.2	73	95.384	2		17.20	25.06/28.38
3802	gi|402753	Translation elongation factor EF-G	5.04	77865.6	89	99.894	14		5.19	92.84/99.06
4202	gi|255550363	Groes chaperonin	8.89	26582.2	73	95.384	2		4.46	91.70/86.91
**Peptidase enzymes**										
2807	gi|9759033	Acyl-peptide hydrolase-like	5.08	76116.9	160	100	10		5.33	92.74/65.70
2905	gi|297742722	Oligopeptidase B	5.21	79684.1	125	100	9		2.16	68.83/56.15
3807	gi|307136309	Serine-type endopeptidase	5.15	83277.2	145	100	13		2.55	84.32/37.22
3901	gi|297742722	Oligopeptidase B	5.21	79684.1	125	100	9		1.55	77.53/44.01
4801	gi|225468332	Similar to oligopeptidase A	5.61	58732.5	81	99.234	9		2.20	99.63/62.75
4802	gi|255572579	Oligopeptidase A, putative	5.71	88118.8	129	100	13		2.13	99.48/44.81
4903	gi|255537515	Aminopeptidase, putative	6.04	98135.3	131	100	15		11.40	98.35/65.03
5902	gi|255537515	Aminopeptidase, putative	6.04	98135.3	304	100	15		10.90	98.02/46.32
5903	gi|25083482	Putative aminopeptidase	5.43	99495.2	252	100	15		15.04	98.38/77.61
5904	gi|255537515	Aminopeptidase, putative	6.04	98135.3	250	100	18		13.49	99.19/50.26
**Protein phosphatase**										
0303	gi|15240071	Putative protein phosphatase 2C 80	7.6	44302	72	94.7	3		2.46	29.15/49.70
**Antioxidation proteins**										
1402	gi|18874402	Galactinol synthase	4.81	38608	130	100	5		7.62	93.74/14.20
1802	gi|11559422	Disulfide isomerase	5.07	37249	589	100	20		3.84	89.41/74.09
1803	gi|11559422	Disulfide isomerase	5.07	37249	589	100	20		3.38	85.47/57.64
1805	gi|11559422	Disulfide isomerase	5.07	37249	589	100	20		3.01	91.93/51.20
1806	gi|11559422	Disulfide isomerase	5.07	37249	589	100	20		3.47	94.47/68.43
2105	gi|240252434	NifS-like protein	6.24	67894.1	69	89.179	10		4.07	11.20/34.32
2107	gi|297842615	Glutathione S-transferase	5.76	75307.3	101	99.993	8		2.22	67.69/9.46
3808	gi|341579690	Betaine-aldehyde dehydrogenase	5.25	55339.3	602	100	16		4.58	77.64/59.24
5804	gi|124057819	Raffinose synthase	5.42	87904.5	96	99.975	17		391.29	99.03/60.22
7204	gi|117663160	Carbonic anhydrase	6.3	10976.5	175	100	5		225.27	80.50/99.93
7302	gi|15222954	Thioredoxin-like protein CDSP32	8.65	33948.5	253	100	8		1.59	26.05/52.03
8202	gi|117663160	Carbonic anhydrase	6.3	10976.5	257	100	7		6.96	71.19/80.24
**Others**										
3803	gi|224065421	Peptidylprolyl isomerase	5.03	64379.7	105	99.997	9		5.19	94.72/75.20
5703	gi|108710583	Adenylosuccinate synthetase	9.07	51473.6	247	100	7		3.04	70.41/61.06
5711	gi|255578102	Imidazole glycerol phosphate synthase subunit hisf	6.62	65225.3	496	100	16		1.88	55.65/64.14
5803	gi|9759324	4-hydroxy-3-methylbut-2-en-1-yl diphosphate synthase	5.89	80394.2	700	100	25		3.81	99.30/74.98
5812	gi|225448296	PREDICTED: hypothetical protein	6.55	106446.4	183	100	10		1175.24	75.18/83.99
7406	gi|18401429	N-carbamoylputrescine amidase	5.71	33683	242	100	6		6.86	92.78/63.85
7606	gi|255562088	Transaminase mtnE	6.95	50909.2	346	100	10		1.77	54.52/42.84

### ROS-related proteins identified by MALDI-TOF-TOF MS

NADPH oxidase, which transfers an electron from NADPH to O_2_ is the principal source of ROS in a short time. In animal cells, the pentose phosphate pathway is the main source of NADPH accumulation [24]. In plant cells, the large amount of NADPH generates from both photosynthesis and pentose phosphate pathway [25]. In this study, we have identified the increase of several photosynthesis and pentose phosphate pathway proteins, which supplied enough NADPH for generating ROS by NADPH oxidase. An important protein, phosphatase PP2C, was identified in our study. Seventy-six PP2C-type phosphatase candidates were identified in *Arabidopsis* and divided into 10 groups [26]. The well-studied PP2C genes, *ABI1* and *ABI2*, are associated with ABA signaling, which involves the closing of stomata by activated I_Ca_ Ca^2+^ Channels induced by ROS [27]. The two genes act differently: *abi1-1* interrupted NADPH oxidase-related ROS generation and *abi2-1* affected the activation of downstream I_Ca_ Ca^2+^ Channels [28]. In our study, PP2C 80 was identified as a glucohexaose-induced ROS-related protein, which may function either in processes related to ROS generation or in signal output, which should be investigated in a future study.

As ROS are toxic for pathogens and for plants themselves, plants must have antioxidation mechanisms for protecting themselves while still killing the pathogens. In our study, we identified eight antioxidation proteins: galactinol synthase, raffinose synthase, NifS-like protein, thioredoxin-like protein CDSP32, disulfide isomerase, glutathione S-transferase, betaine-aldehyde dehydrogenase and carbonic anhydrase. Galactinol synthase and raffinose synthase are important enzymes for the synthesis of the raffinose family of oligosaccharides, which are important for protecting plants during the stress response [29-31]. Nishizawa et al. found that galactinol and raffinose protected plants from oxidative stress by removing scavenging hydroxyl radicals [30]. The active center of thioredoxins comprises four amino acids, Cys-Gly-Pro-Cys, which can reduce disulfide bridges to protect plants from oxidative damage [32]. We identified a 32KDa protein thioredoxin, CDSP32. Rey et al. found that the six targets of the overexpression mutants of thioredoxin CDSP32 are involved in a strong resistance to oxidative stress. As a result, compared with other proteins in the thioredoxin family, thioredoxin CDSP32 is presumed to function mainly in antioxidative stress [33]. The reduction of disulfide bridges of thioredoxins is accompanied by an iron-sulfur cluster [34] and another identified protein, NifS-like protein, is important for iron-sulfur cluster synthesis [35]. In addition, the disulfide bridges of thioredoxins are catalyzed by an important chaperone, disulfide isomerase, which was also identified in our research [36]. We identified another three proteins in our research, glutathione-S-transferase, betaine-aldehyde dehydrogenase and carbonic anhydrase. These three are all antioxidative stress proteins, functioning through glutathione [37], betaine [38] and radical scavenging, respectively [39]. These antioxidative stress proteins are further evidence for the induction of ROS accumulation by glucohexaose treatment and may represent the plant protective mechanism induced during oxidative stress.

## Discussion

ROS signaling play an important role in resistance to pathogens as signal molecules in large variety of plant species. In this study, we noticed both H_2_O_2_ and O^2-^ accumulation in cucumber cotyledons is a part of glucohexaose induced resistance. Normally, there are two phases of ROS accumulation induced by pathogen but only one phase of ROS accumulation induced by elicitors [40]. The glucohexaose elicitor shares similar mechanism with elicitors, which accumulated shortly after treatment and reached the peak level at about 5 h after treatment. DPI can totally repress the generation of H_2_O_2_ and O^2-^ and proteomic analysis showed the increasing level of enzymes is relevant to synthesis of NADPH. As a result, the generation of cucumber ROS induced by glucohexaose elicitor is mainly produced by NADPH oxidase. NADPH oxidase appeared from moss and strongly expanded in vascular plants [16]. Our study indicates similar mechanism of ROS generation in cucumber.

We have identified an interesting protein PP2C 80 in our study. It is well known that PP2Cs are important negative regulators in ABA signaling. ABA is a hormone involving stress tolerance. ABA can induce stomata closing and ROS generation but NADPH oxidase double mutant *atrbohD/F* cannot [41]. The activity of two famous PP2Cs ABI1 and ABI2 are repressed by H_2_O_2_ in *Arabidopsis*[42,43]. In our study, the level of PP2C 80 increased after H_2_O_2_ accumulation. We have no idea whether the PP2C is involved in ABA signaling and why it accumulate after H_2_O_2_ accumulation. But it is an indication that glucohexaose induced resistance may have relationship with ABA signaling.

The identification of thioredoxin could contribute to the regulation of ROS level. Scavengers glutathione-S-transferase, betaine-aldehyde dehydrogenase and carbonic anhydrase are increased after gluchexaose treatment. Thioredoxin also increase after ROS accumulation in *Arabidopsis*, soybeans and potatoes [44-46]. Our study showed similar mechanism in cucumber. Meanwhile, SOD, POD and APX activities are increased in our study. The scavengers of ROS may function after ROS accumulation to prevent further damage to plant cells. We also found scavengers CAT and GPX decreased after glucohexaose treatment. They may have functions in ROS accumulation.

## Concluding remarks

Our study detected the accumulation of ROS is a part of mechanism of glucohexaose induced resistance in cucumber cotyledons. NADPH oxidase is in charge of the main generation of the rapidly output of ROS. ROS scavengers’ activities change in the progress to regulate ROS level. Thirty-seven up-regulate proteins were identified after glucohexaose treatment and repressed by DPI, which are involved in photosynthesis, respiration, translation, phosphorylation and antioxidation. PP2C might play a crucial role in processes related to ROS generation and signal transduction, and antioxidation proteins increased after glucohexaose treatment, indicating the involvement of a self-protection mechanism in the process. It will be interesting to find out the regulatory mechanism underlying the induction of ROS-targeting proteins via ROS, and provide clues concerning the mechanism of glucohexaose-induced resistance and a theoretical basis for developing safe farm chemicals for vegetable production.

## Abbreviations

H_2_O_2_: Hydrogen peroxide; O^2-^: Superoxide; DPI: Diphenyleneiodonium chloride; DMTU: Dimethylthiourea; SOD: Superoxide dismutase; CAT: Catalase; APX: Ascorbate peroxidase; GPX: Glutathione peroxidase; MDA: Malonaldehyde; POD: Peroxidase; DAB: Diaminobenzidine; ABA: Abscisic acid; SA: Salicylic acid; JA: Jasmonic acid; TCA: Trichloroacetic acid; TBP: Tributyl phosphate; P6: Glucohexaose.

## Competing interests

The authors declare that they have no competing interests.

## Authors' contributions

HF is responsible for experimental design and manuscript revising. YH measured the H_2_O_2_ and O^2-^ contents; run the 2D gels; analyzed 2D image and MS data. NL and CL measured the contents of scavenger enzymes activity. YY and SC is responsible for mass spectrometry analysis. All authors read and approved the final manuscript.

## Supplementary Material

Additional file 1: Figure S1 2D maps of cucumber cotyledon proteins with different treatment and the spots identified. **A**, Control group, fraction F1; **B**, the cucumber cotyledons treated with 50 μg/mL glucohexaose for five hours, fraction F1; **C**, Before treated with 50 μg/mL glucohexaose, DPI were incubated for four hours, fraction F1; **D**, Before treated with 50 μg/mL glucohexaose, DMTU were incubated for four hours, fraction F1; **E**, Control group, fraction F2; **F**, the cucumber cotyledons treated with 50 μg/mL glucohexaose for five hours, fraction F2; **G**, Before treated with 50 μg/mL glucohexaose, DPI were incubated for four hours, fraction F2; **H**, Before treated with 50 μg/mL glucohexaose, DMTU were incubated for four hours, fraction F2. **Figure S2.** Differential protein spots and their relative abundance. All identified protein spots’ Representative differential protein spots and their relative abundance information. Click here for file
