# Supplementary material for: Proteomic analysis of Cucumis sativus cotyledons after glucohexaose treatment as a part of ROS accumulation related resistance mechanism
Source: Proteome Sci. 2014 Jun 17;12:34. doi: 10.1186/1477-5956-12-34 (PMC4098955; doi:10.1186/1477-5956-12-34)
Supplement: Additional file 1: Figure S1 — 2D maps of cucumber cotyledon proteins with different treatment and the spots identified. A, Control group, fraction F1; B, the cucumber cotyledons treated with 50 μg/mL glucohexaose for five hours, fraction F1; C, Before treated with 50 μg/mL glucohexaose, DPI were incubated for four hours, fraction F1; D, Before treated with 50 μg/mL glucohexaose, DMTU were incubated for four hours, fraction F1; E, Control group, fraction F2; F, the cucumber cotyledons treated with 50 μg/mL glucohexaose for five hours, fraction F2; G, Before treated with 50 μg/mL glucohexaose, DPI were incubated for four hours, fraction F2; H, Before treated with 50 μg/mL glucohexaose, DMTU were incubated for four hours, fraction F2. Figure S2. Differential protein spots and their relative abundance. All identified protein spots’ Representative differential protein spots and their relative abundance information. [file 1477-5956-12-34-S1.docx]

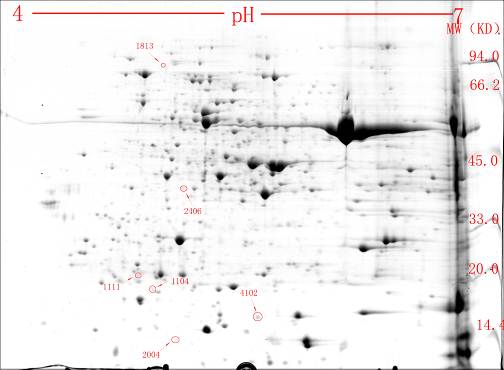

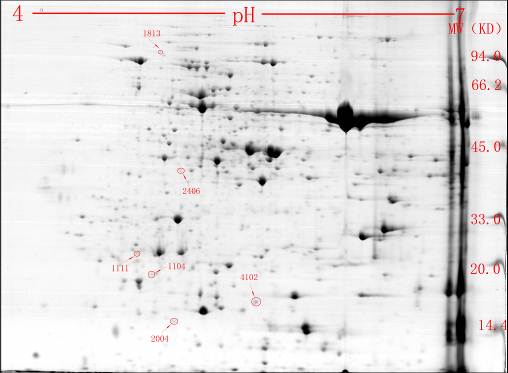


**A B**


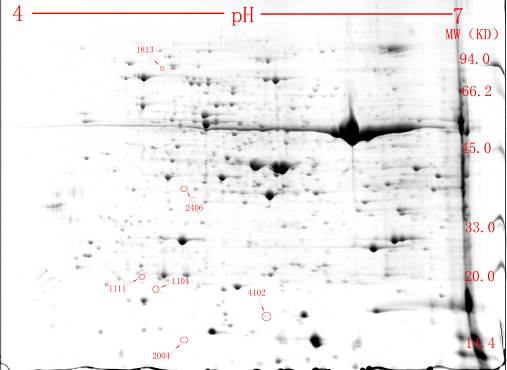

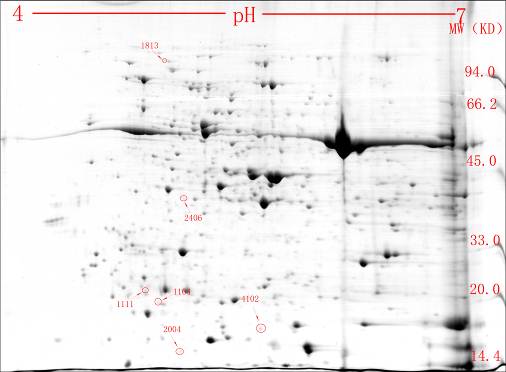


C D


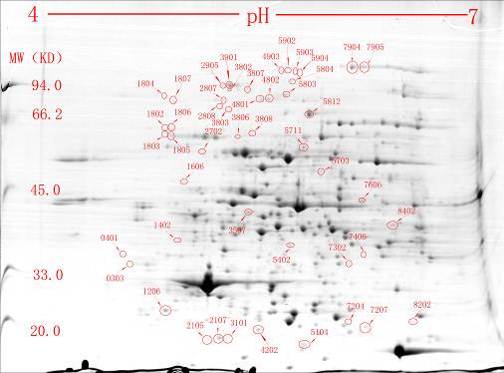

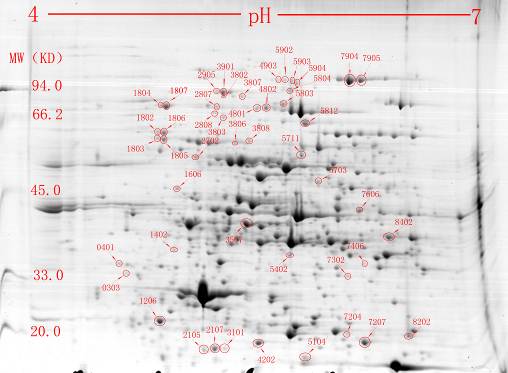


E F


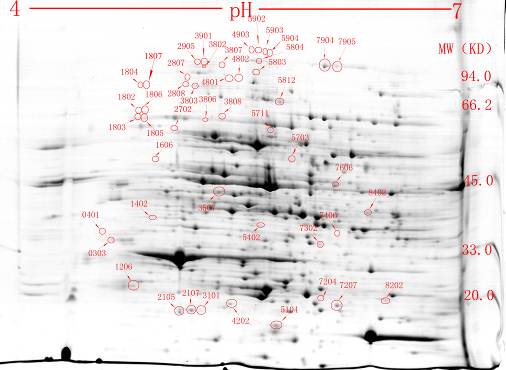

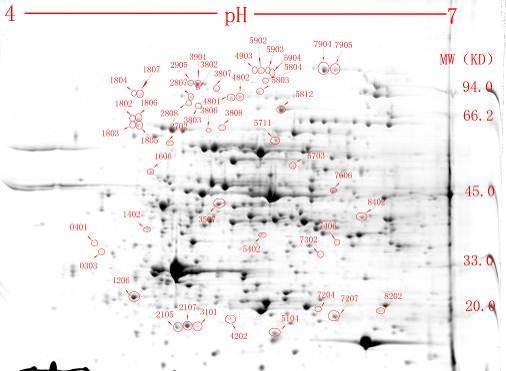


G H

Fig. S1 2D maps of cucumber cotyledon proteins with different treatment and the spots identified

A, Control group, fraction F1; B, the cucumber cotyledons treated with 50μg/mL glucohexaose for five hours, fraction F1; C, Before treated with 50μg/mL glucohexaose, DPI were incubated for four hours, fraction F1; D, Before treated with 50μg/mL glucohexaose, DMTU were incubated for four hours, fraction F1; E, Control group, fraction F2; F, the cucumber cotyledons treated with 50μg/mL glucohexaose for five hours, fraction F2; G, Before treated with 50μg/mL glucohexaose, DPI were incubated for four hours, fraction F2; H, Before treated with 50μg/mL glucohexaose, DMTU were incubated for four hours, fraction F2.


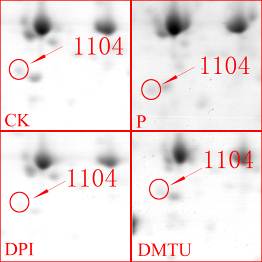

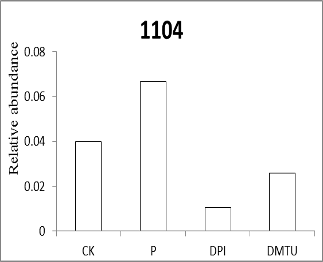

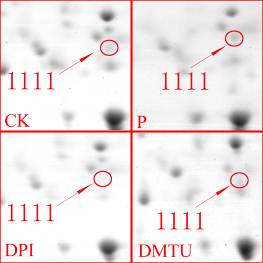

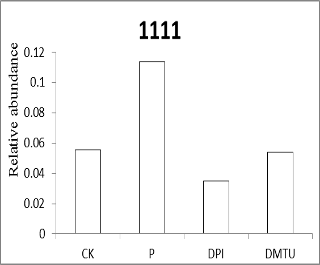


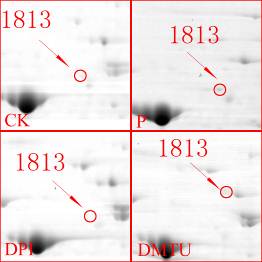

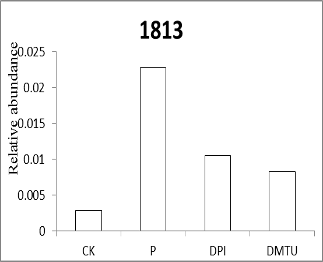

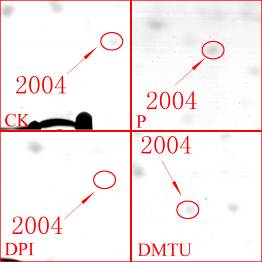

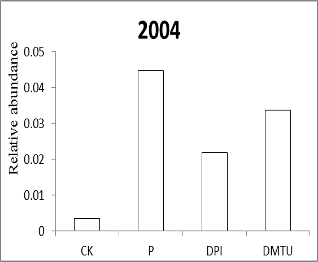


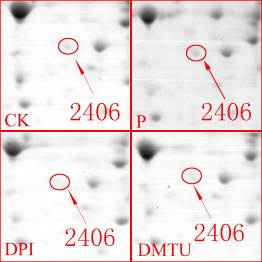

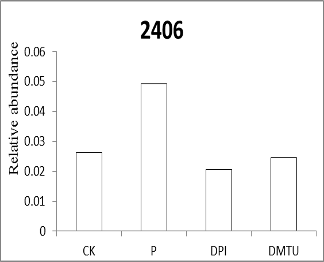

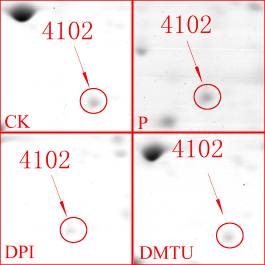

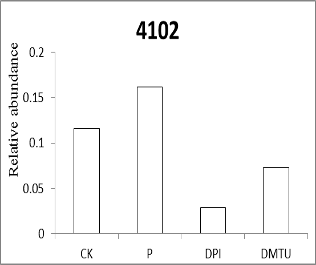


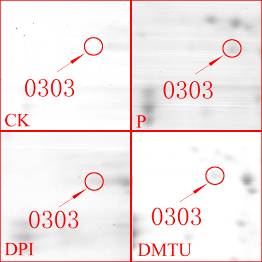

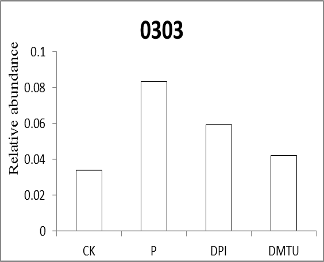

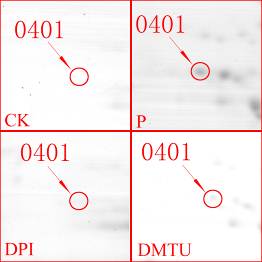

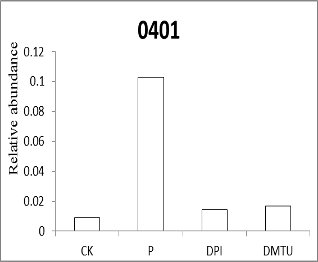


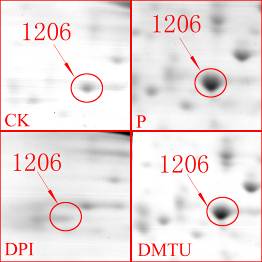

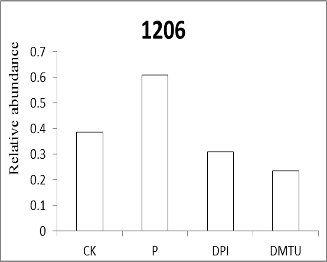

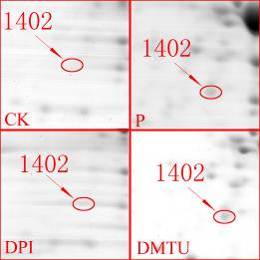

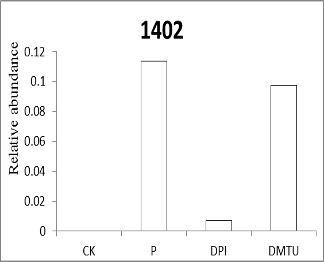


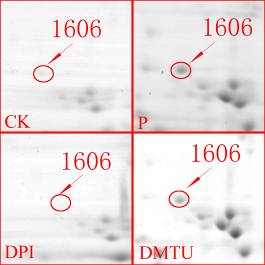

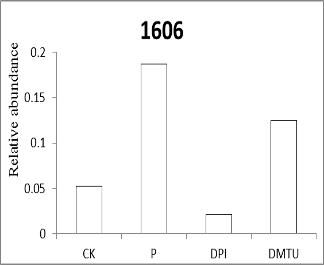

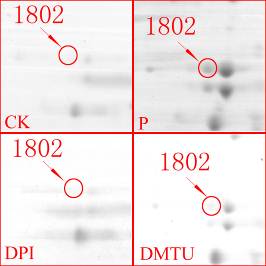

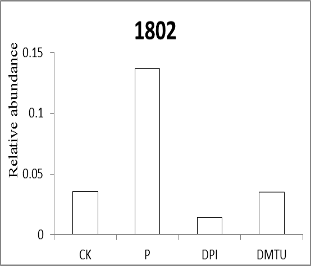


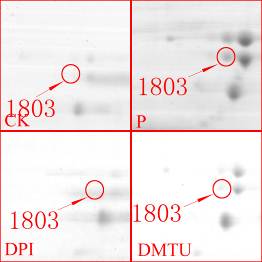

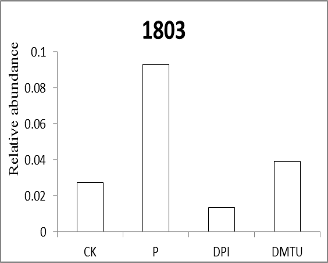

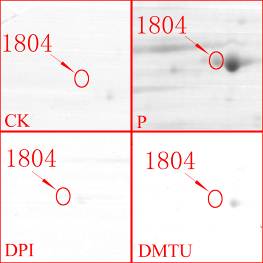

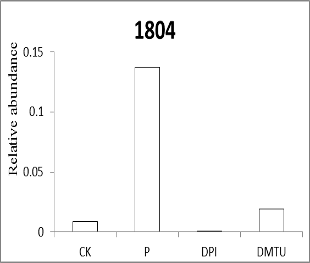


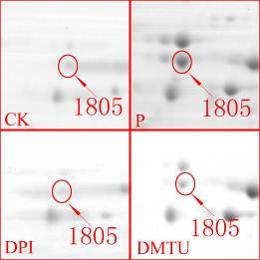

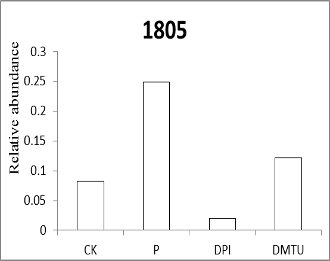

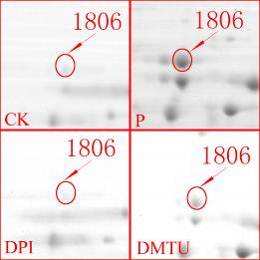

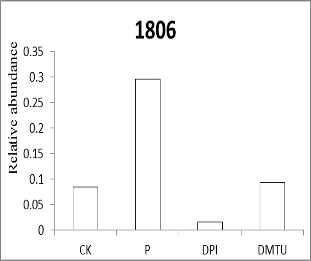


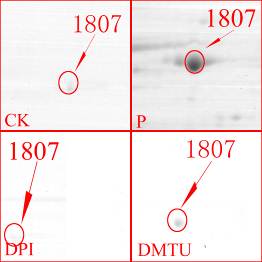

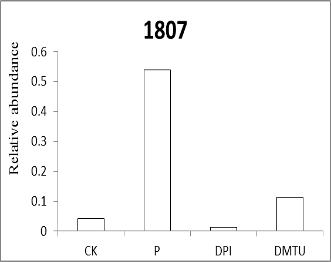

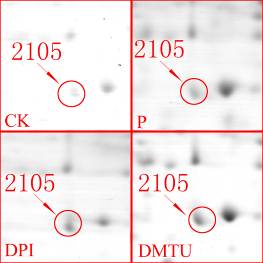

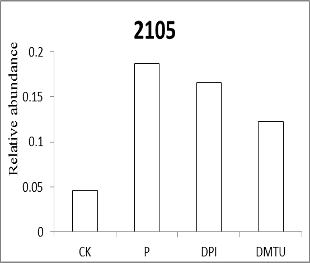


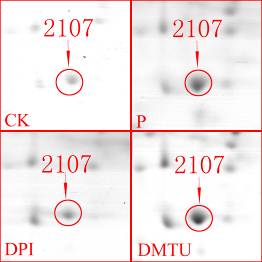

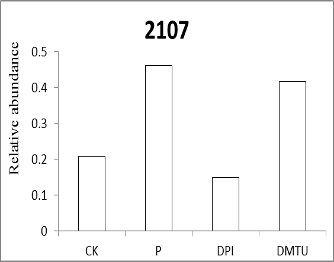

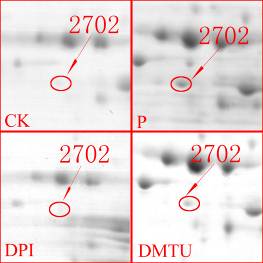

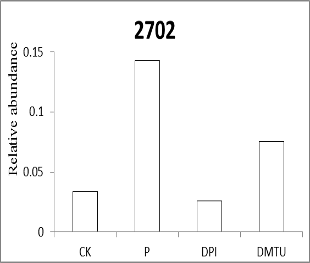


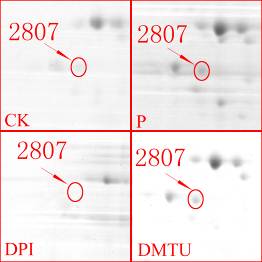

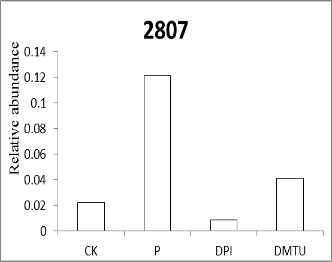

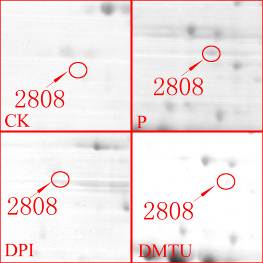

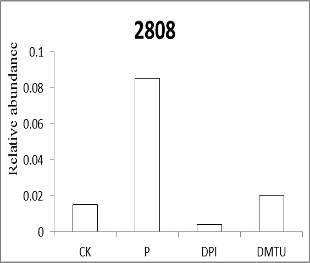


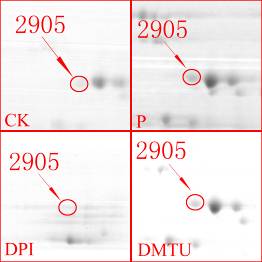

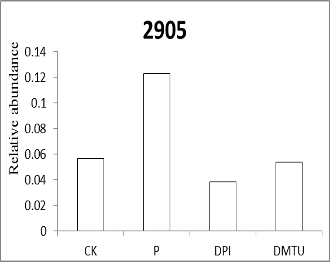

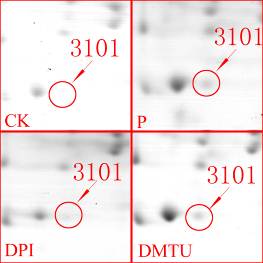

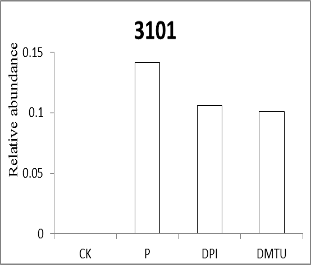


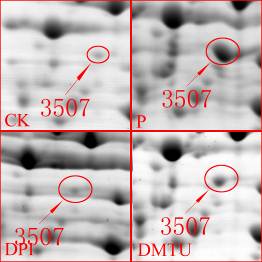

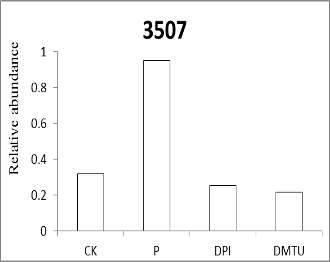

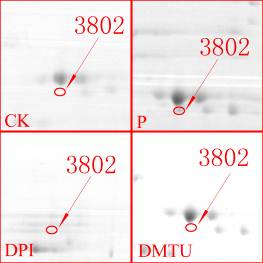

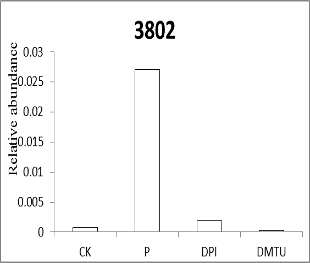


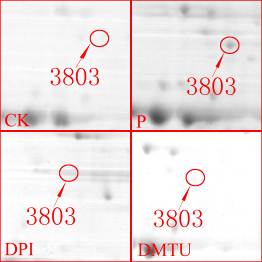

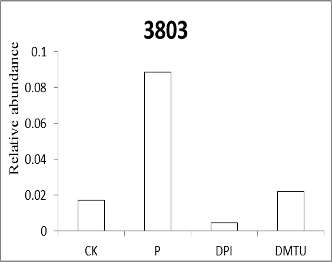

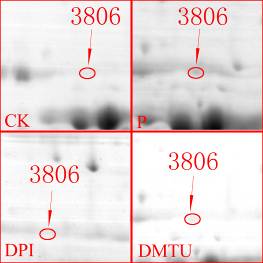

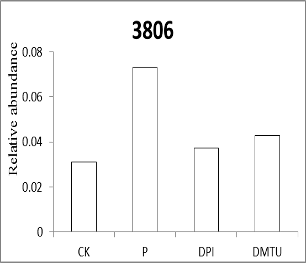


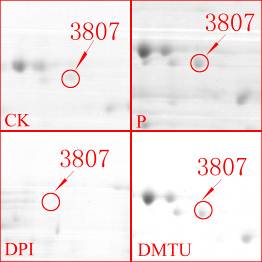

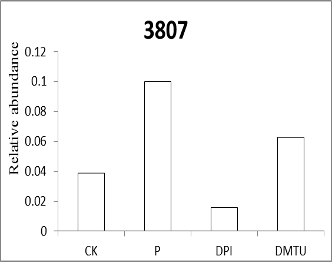

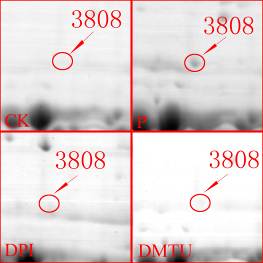

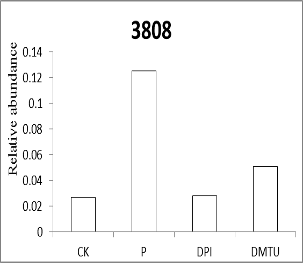


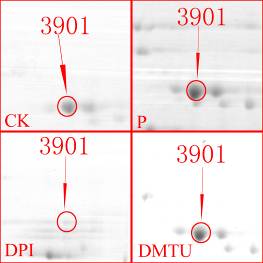

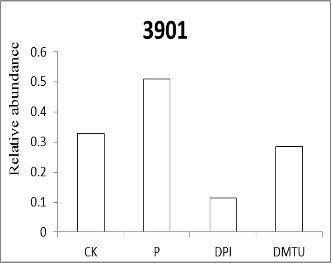

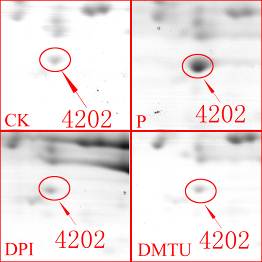

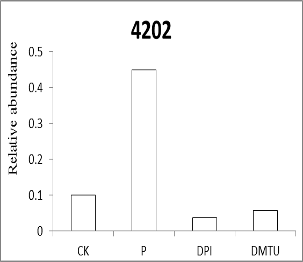


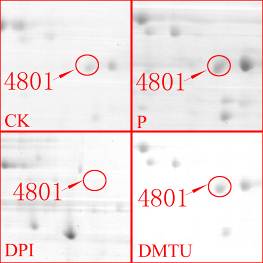

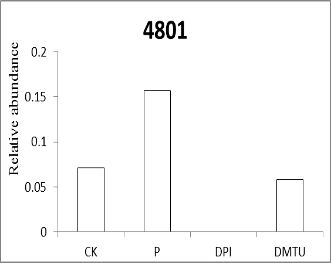

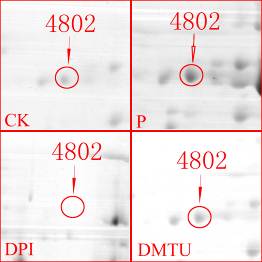

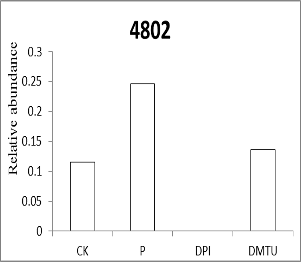


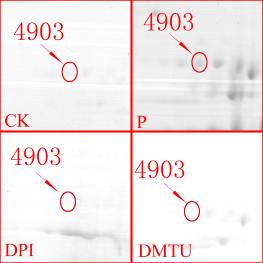

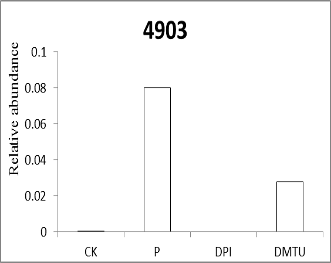

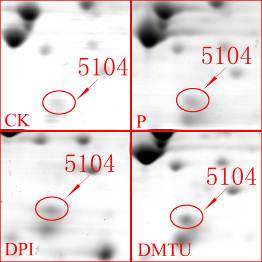

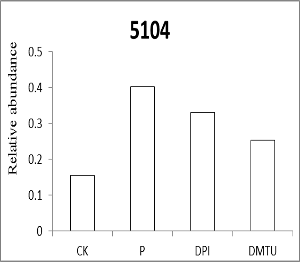


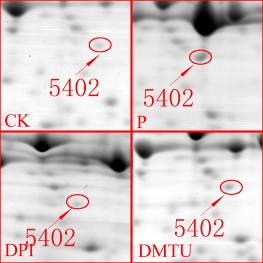

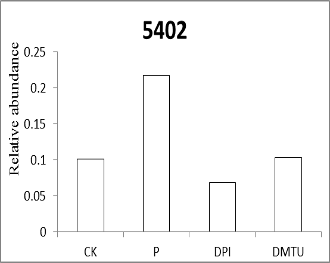

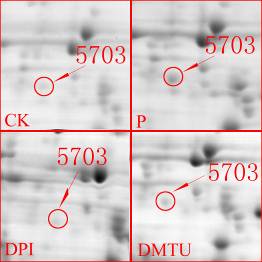

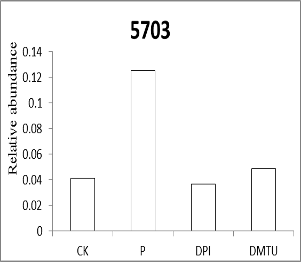


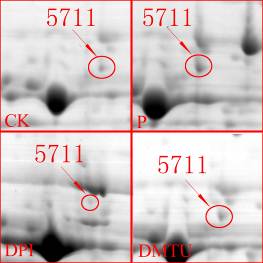

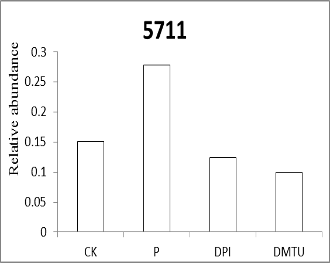

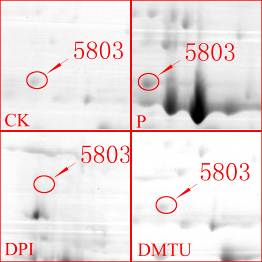

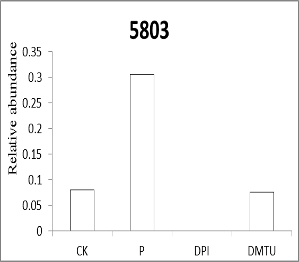


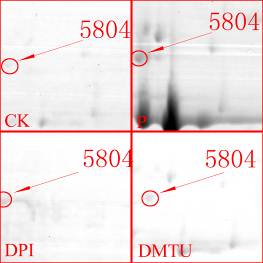

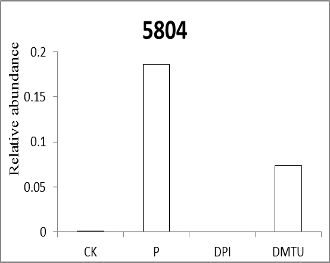

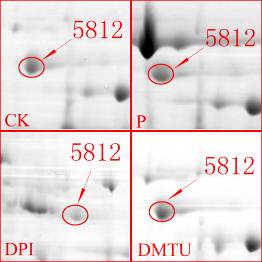

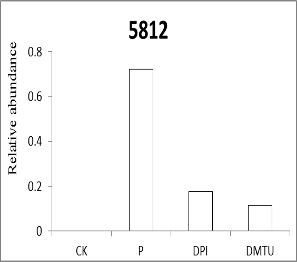


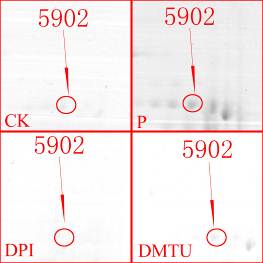

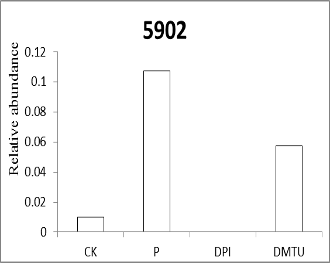

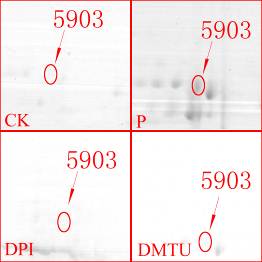

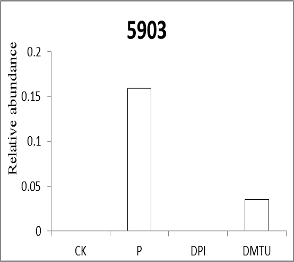


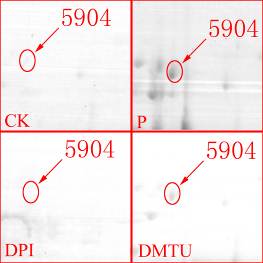

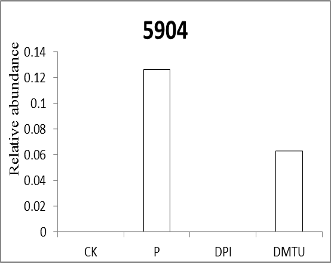

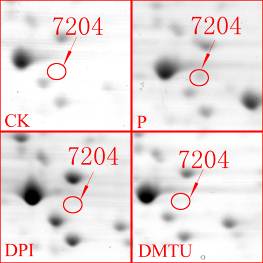

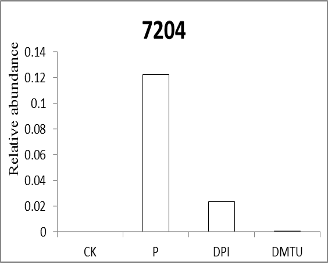


Fig. S2 Differential protein spots and their relative abundance

All identified protein spots’ Representative differential protein spots and their relative abundance information.
